# Successful Nonsurgical Treatment of a Radial Artery Pseudoaneurysm Following Transradial Coronary Angiography

**Published:** 2017-04

**Authors:** Reza Ghanavati, Mehran Arab Ahmadi, Behdad Behnam

**Affiliations:** 1 *Rasoul Akram General Hospital, Iran University of Medical Sciences, Tehran, Iran* *.*; 2 *Functional Neurosurgery Research * *Center, * *Shahid Beheshti University of Medical Sciences, Tehran, Iran.*

**Keywords:** *Coronary angiography*, *Angioplasty*, *Complications*, *Aneurysm, false*

## Abstract

Transradial coronary angiography has been known as an alternative to the transfemoral approach with fewer serious complications. Radial artery pseudoaneurysms present as a rare complication of transradial catheterization. Although some methods have been applied for the obliteration of pseudoaneurysms, the use of radial bands such as the TR Band^®^ (Terumo Medical Corporation, Somerset, NJ) is a novel efficient technique only suggested by a few reports. We describe a 34-year-old man, who underwent transradial primary coronary angiography due to ST-elevation myocardial infarction. Two months later, he noticed a pulsatile mass on his hand where the catheterization was done. Ultrasonography proved the diagnosis of a pseudoaneurysm. Consequently, a TR Band^®^ was applied to compress the mass. Interestingly, 24 hours later, ultrasonography confirmed a thrombosed pseudoaneurysm and the pulsatile mass had completely disappeared gradually without recurrence at 2 months’ follow-up. Hence, this case report aims to propose the TR Band^®^ as an effective noninvasive method for the treatment of pseudoaneurysms following catheterization.

## Introduction

Transradial coronary angiography (TRA) was first suggested by Campeau^   1 ^ more than 20 years ago. Transradial percutaneous coronary intervention has been known as an effective intervention with better outcomes and fewer vascular complications than any other procedure, although it is associated with a few rare complications.^2^ 

Based on previous meta-analyses, major complications in the site of catheterization are seen in 0.3% of the radial approach versus 2.8% of the femoral intervention.^   3 ^ Pseudoaneurysms have been known as a rare complication, affecting 3 per 10000 patients after days to weeks following TRA.^4^ Common manifestations include localized swelling and pain in the wrist, antecubital fossa, or forearm area.^   4 ^ It is usually diagnosed based on physical examination, Doppler ultrasound, or angiography. 

Here in, we report a case of a radial artery pseudoaneurysm, which was treated successfully with the local compression technique (a TR Band^®^). To our knowledge, there are only a few cases of this novel method for the treatment of radial pseudoaneurysms following TRA.

## Case Report

The patient was a 32-year-old man, who referred to our emergency department with the complaint of chest pain. At the time, he was diagnosed with anterior ST-elevation myocardial infarction (STEMI) based on history taking and electrocardiogram (ECG). The patient underwent primary percutaneous coronary intervention on the proximal portion of his left anterior descending artery through the right radial approach with a 6-F hydrophilic radial sheath, a 5-F Merit Performa catheter, and a 6-F guiding catheter. He had an uncomplicated hospital course of stay and on discharge, his echocardiography revealed an ejection fraction of 25%, accompanied by a severely smokey pattern in the left ventricle with anteroseptal wall motion abnormality.

Based on the echocardiographic findings, triple antithrombotic therapy - comprising Aspirin (80 mg daily), clopidogrel (75 mg daily), and warfarin (goal international normalized ratio [INR] = 2) - was started for him.

**Figure 1:**
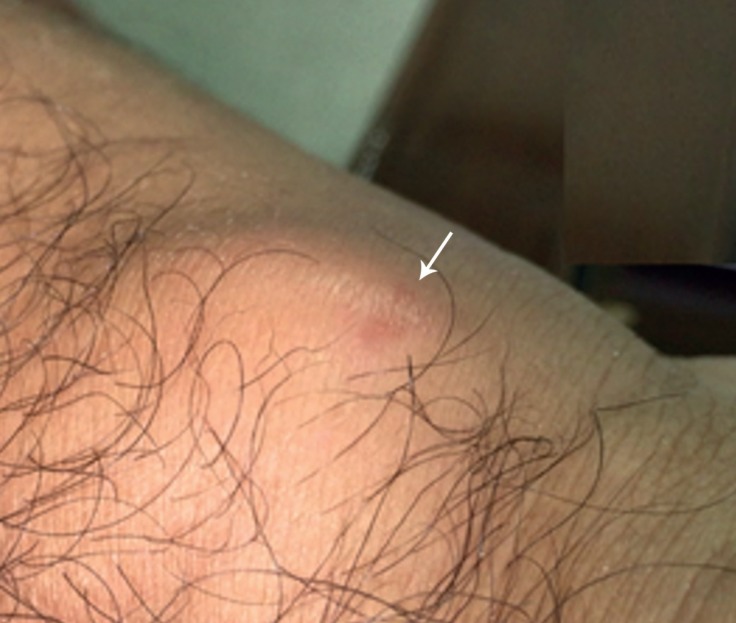
Pulsatile mass (arrow) at the site of catheterization on the right hand (before treatment)

The patient was well at his follow-up visit until 2 months after the first admission, when he developed a pulsatile painful mass in his right wrist (Figure 1A, 1B), which appeared when he was lifting a heavy box. Physical examination was unremarkable, except for the pulsatile wrist mass, about 3 × 3 cm in size, with a normal capillary filling of the 5th finger.

Color Doppler ultrasonography showed a 2.5 × 2 cm pseudoaneurysm with a 4-mm neck and a to-and-fro flow. Warfarin was discontinued, and the pulsatile mass was compressed using a TR Band^®^ (Terumo Medical Corporation, Somerset, NJ) for 24 hours. The TR Band^®^ was placed on his right wrist, just at the site of the pseudoaneurysm and was inflated with 8 cc of air with a normal pulse oximeter waveform at the 5th finger.

**Figure 2:**
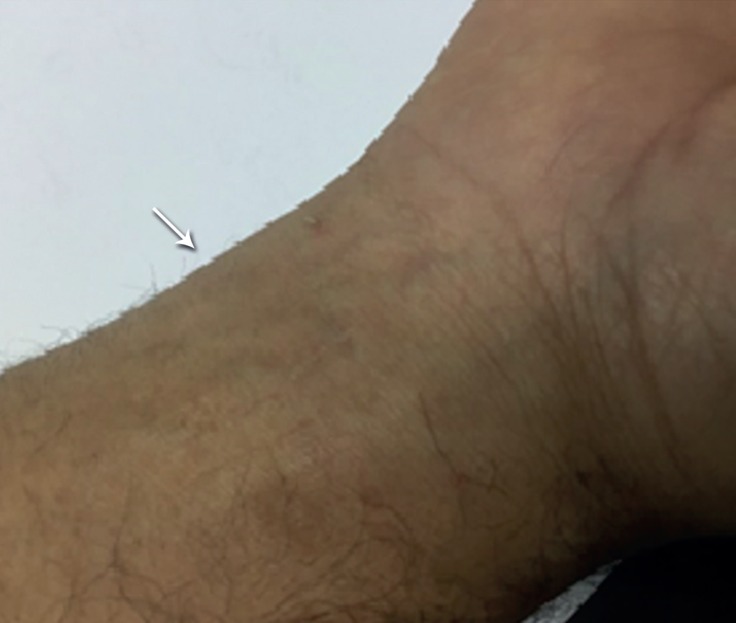
Pseudoaneurysm has completely disappeared (arrow) without recurrence at 2 months’ follow-up on the right hand (after treatment).

During the compression, the patient experienced mild pain in his right hand without any evidence of cyanosis on examination. After the first 24 hours, the pulsations disappeared and follow-up ultrasonography showed a thrombosed pseudoaneurysm without a to-and-fro flow. It completely improved with no relapse at his 2 months’ follow-up (Figure 2).

## Discussion

Herein, we reported a case of a radial artery pseudoaneurysm after cardiac catheterization that was successfully treated through a noninvasive compressive technique with a TR Band^®^. 

Inappropriate catheter sheath size (large size), massive use of antiplatelet agents, infection at the site of intervention, and insufficient postprocedural compression are known as predisposing factors to the development of pseudoaneurysms.^   5 ^ Accordingly, adequate compression at the site of intervention and observation for any sign of pain or swelling after the procedure are very useful in the prevention of pseudoaneurysms. 

In most case reports and also in our patient, the most possible factor in developing pseudoaneurysms was antiplatelet therapy. However, based on the 2013 guideline of the American College of Cardiology/American Heart Association (ACC/AHA)for the management of patients with STEMI, aspirin plus a P2Y12 receptor antagonist and vitamin K antagonist(class of recommendation: IIb, level of evidence: C)should be considered for patients who are diagnosed with anterior STEMI, have undergone percutaneous coronary intervention, and have anterior wall motion abnormalities on echocardiography (as was seen in our patient),^   6 ^ particularly in cases with a low risk of bleeding.

Currently, the available proven techniques for the treatment of pseudoaneurysms include surgical repair, ultrasound-guided compression repair (UGCR), and ultrasound-guided thrombin injection (UGTI).^7^

Along with the development of nonoperative minimally invasive methods, surgery for repairing post-catheterization pseudoaneurysms has gradually become less important.^   8 ^ UGCR is a time-consuming technique that includes 10-minute intervals until the obliteration of the pseudoaneurysm.^   9 ^

Recently, UGTI has provided a new alternative method with higher success and rapid response. In this procedure, thrombin is injected directly to the pseudoaneurysm sac under ultrasound guide.^10^ However, it is probable that thrombin escapes from the pseudoaneurysm, which can occlude the distal vessels and cause pain, paresthesia, and skin necrosis.^   10 ^

Beside common conventional treatments, a novel strategy by applying external compression has been suggested as a cost-effective and simple method that can be considered a first-line treatment for patients with small-to-moderate-sized radial arteries.^   11 ^

During the recent years, a few case reports have applied different devices for external compression in radial artery treatment for pseudoaneurysms. Accordingly, the TR Band^®^ has been designed for external compression with 2 adjustable, inflatable balloons on the radial artery. Although it compresses the radial artery, it causes neither local nerve compression nor venous stasis.^   12 ^

For the first time, Liouet al.^11^ in 2010 reported successful treatment of radial artery pseudoaneurysms by using the TR Band^®^. Four years later, Cauchiet al.^12^ suggested the TR Band^®^ for a post-catheterization pseudoaneurysm in a 45-year-old patient, who developed a pseudoaneurysm following TRA.

On the other hand, except for the TR Band^®^, other devices with a similar mechanism have been found useful by a few reports. In this regard, Nazeret al.^13^ used the HemoBand (Hemoband Corporation), which showed some relapses.

To our knowledge, this is a rare report of the successful treatment of a radial pseudoaneurysm in the site of catheterization with a TR Band® in a young man. The pseudoaneurysm completely disappeared without any complication within a 2-month follow-up period.

## Conclusion

This case is presented to encourage cardiologists to consider the TR Band® as an advantageous, noninvasive, and cost-effective device for the treatment of pseudoaneurysms, especially in patients who have received anticoagulant therapy. 

## Notes:

***This paper should be cited as:*** Ghanavati R, Arab Ahmadi M, Behnam B. Successful Nonsurgical Treatment of a Radial Artery Pseudoaneurysm Following Transradial Coronary Angiography. J Teh Univ Heart Ctr 2017;12(2):82-84.

## References

[1] Campeau L (1989). Percutaneous radial artery approach for coronary angiography. Cathet Cardiovasc Diagn.

[2] Shroff A, Siddiqui S, Burg A, Singla I (2013). Identification and management of complications of transradial procedures. Curr Cardiol Rep.

[3] Agostoni P, Biondi-Zoccai GG, de Benedictis ML, Rigattieri S, Turri M, Anselmi M, Vassanelli C, Zardini P, Louvard Y, Hamon M (2004). Radial versus femoral approach for percutaneous coronary diagnostic and interventional procedures: systematic overview and meta-analysis of randomized trials. J Am Coll Cardiol.

[4] Blasco A, Oteo JF, Fontanilla T, Salamanca J, Ocaranza R, Goicolea J (2005). Unusual complications of cardiac catheterization via the radial artery. Rev Esp Cardiol.

[5] Collins N, Wainstein R, Ward M, Bhagwandeen R, Dzavik V (2012). Pseudoaneurysm after transradial cardiac catheterization: case series and review of the literature. Catheter Cardiovasc Interv.

[6] American College of Emergency Physicians, Society for Cardiovascular Angiography, Interventions, O'Gara PT, Kushner FG, Ascheim DD, Casey DE Jr, Chung MK, de Lemos JA, Ettinger SM, Fang JC, Fesmire FM, Franklin BA, Granger CB, Krumholz HM, Linderbaum JA, Morrow DA, Newby LK, Ornato JP, Ou N, Radford MJ, Tamis-Holland JE, Tommaso CL, Tracy CM, Woo YJ, Zhao DX, Anderson JL, Jacobs AK, Halperin JL, Albert NM, Brindis RG, Creager MA, DeMets D, Guyton RA, Hochman JS, Kovacs RJ, Kushner FG, Ohman EM, Stevenson WG, Yancy CW (2013). 2013 ACCF/AHA guideline for the management of ST-elevation myocardial infarction: a report of the American College of Cardiology Foundation/American Heart Association Task Force on Practice Guidelines. J Am Coll Cardiol.

[7] Mohamed MO, Saif M, Townend JN, Khan SQ (2015). Successful treatment of a radial artery pseudoaneurysm in an octogenarian. BMJ Case Rep.

[8] Bhat T, Teli S, Bhat H, Akhtar M, Meghani M, Lafferty J, Gala B (2012). Access-site complications and their management during transradial cardiac catheterization. Expert Rev Cardiovasc Ther.

[9] Joseph V, Sambhaji C, Prakashini K (2013). Radial artery pseudoaneurysm managed by prolonged ultrasound-guided compression repair and aided by interval application of compression device. Australas Med J.

[10] Pozniak MA, Mitchell C, Ledwidge M (2005). Radial artery pseudoaneurysm: a maneuver to decrease the risk of thrombin therapy. J Ultrasound Med.

[11] Liou M, Tung F, Kanei Y, Kwan T (2010). Treatment of radial artery pseudoaneurysm using a novel compression device. J Invasive Cardiol.

[12] Cauchi MP, Robb PM, Zemple RP, Ball TC (2014). Radial artery pseudoaneurysm: a simplified treatment method. J Ultrasound Med.

[13] Nazer B, Boyle A (2013). Treatment of recurrent radial artery pseudoaneurysms by prolonged mechanical compression. J Invasive Cardiol.

